# Validation of Urinary Glycosaminoglycans in Iranian patients with Mucopolysaccharidase type I: The effect of urine sedimentation characteristics

**Published:** 2014

**Authors:** Mohammad ABDI, Mohammad Said HAKHAMANESHI, Mohammad Reza ALAEI, Namam-Ali AZADI, Rahim VAKILI, Daniel ZAMANFAR, Mohammad TAGHIKHANI, Shohreh KHATAMI

**Affiliations:** 1.Department of Clinical Biochemistry, Faculty of Medical Sciences, Tarbiat Modares University, Tehran, Iran; 2.Department of Biochemistry and Nutrition, Faculty of Medicine, Kurdistan University of Medical Sciences, Sanandaj, Iran; 3 Department of Pediatric, Faculty of Medicine, Shahid Beheshti University of Medical Sciences, Tehran, Iran; 4.Department of Epidemiology and Biostatistics, Faculty of Medicine, Kurdistan University of Medical Sciences, Sanandaj, Iran; 5 Department of Pediatrics, Imam Reza Hospital, Mashhad University of Medical Sciences, Mashhad, Iran; 6 Department of Pediatric, Faculty of Medicine, Mazandaran University of Medical Sciences, Sari, Iran; 7 Department of Biochemistry, Pasteur Institute of Iran, Tehran, Iran

**Keywords:** Mucopolysaccharidosis type I, Dimethyl methylene blue test, Berry spot test, Glycosaminoglycans, Diagnostic value, Iran

## Abstract

**Objective:**

The first line-screening test for mucopolysaccharidosis is based on measurement of urinary glycosaminoglycans. The most reliable test for measurement of urine glycosaminoglycans is the 1,9-dimethyleneblue colorimetric assay. Biological markers are affected by ethnical factors, for this reason, the World Health Organization recommends that the diagnostic test characteristics should be used to determine results for different populations. This study determines the diagnostic value of 1,9-dimethyleneblue tests for diagnosis of mucopolysaccharidosis type I patients in Iran.

**Materials & Methods:**

In addition to routine urine analysis, the qualitative and quantitative measurements of urine glucosaminoglycans were performed with the Berry spot test and 1,9-dimethyleneblue assay. Diagnostic values of the tests were determined using the ROC curve.

**Results:**

Urine total glycosaminoglycans were significantly higher in male subjects than in female subjects. Glycosaminoglycan concentration was markedly decreased in specimens with elevated white blood cell and epithelial cells count. Using a cut-off level of 10.37 mg/g creatinine, sensitivity, and specificity were 100% and 97.22%, respectively, for a 1,9-dimethyleneblue colorimetric assay.

**Conclusion:**

Urine glycosaminoglycans concentration significantly differs in our studied population. In addition to determine diagnostic validity of the 1,9-dimethyleneblue test, our results demonstrate the usefulness of measuring glycosaminoglycans for early screening of mucopolysaccharidosis type I Iran.

## Introduction

Glycosaminoglycans (GAGs) are composed of long sugar chains containing repeating units of highly sulfated, alternating hexuronic acid, and hexosamine residues. Keratan sulfate (KS), heparan sulfate (HS), dermatan sulfate (DS), and chondroitin sulfate (CS) are the main types of GAGs (1-4). GAGs are important components of plasma membranes and the extra-cellular matrix (5,6).

GAG metabolic abnormality is due to enzyme deficiency results in the progressive accumulation of the molecules and leads to an imperceptible deterioration of cells, tissues, and organs that may lead to the onset of a mucopolysaccharidosis (MPS) clinical phenotype (7,8). There are 11 enzyme deficiencies that result in seven distinct MPS clinical syndromes and their subtypes. Mucopolysaccharidosis type I (MPSI, Mc Kusick 252800) is an inherited autosomal recessive disease that results from a deficiency of α-L-iduronidase (IDUA; EC3.2.1.76) activity and results in progressive accumulation of dermatan and heparan sulfate, in lysosomes that may cause the clinical phenotype within a spectrum of severity ranging from severe Hurler syndrome to relatively mild Scheie syndrome (8-12).

Definitive diagnosis of a specific type of the MPS syndrome is based on enzyme analysis (13). It is not reasonable to check all 11 enzymes for every suspected patient. A simple screening test, along with clinical features, is needed to limit the list of potential patients and enzymes to be assayed (14). It is known that all MPS lead to increased urinary GAGs excretion. Therefore, a first line-screening assay for MPSs is a complete quantitative and qualitative analysis of urinary GAGs in suspected patients to avoid tedious and expensive further laboratory testing of patients (7,15). Numerous methods have been developed for this purpose. The Berry spot test provides a rapid qualitative evaluation of urine. GAGs react with toluidine blue, a cationic dye, to yield a purple colored compound (8, 16-18). Today, a spectrophotometric assay using 1,9-dimethyleneblue (DMB) has been developed and is regarded as being more reliable than earlier quantitative methods such as the uronic acid carbazole test (8). Little data exists on diagnostic value of urinary GAGs levels for different ethnic groups and there are no other studies that investigate the role of this marker in the Iranian population. Therefore, in this paper, we study the diagnostic efficiency of urinary GAGs in Iranian MPSI patients to the primary screening of MPSI.

## Materials & Methods

**Subjects**

In this study, all patients were identified as likely cases for MPS from clinical phenotypes between January 2011 and October 2013 were examined for MPS. From this, we identified 15 subjects who were diagnosed as MPSI patients. Diagnosis of MPSI was male by fluorometric measurement of alpha-L-Iduronidase activity in dried blood spot samples. A total of 185 cases (ages: 1-month to 18 years of age) who were admitted to Besat Hospital in Sanandaj (Kurdistan, Iran) were enrolled in the study as the control group. 

Written informed consent was obtained and the project was approved by the Research Ethics Committee of Tarbiat Modares University (Tehran, Iran). Patients who were being treated with Laronidase were excluded from this study. All subjects were categorized into 6 groups according to age and named as group 1 (less than 1 years old), group 2 (1–2 years old), group 3 (2–5 years old), group 4 (5–9 years old), and group 5 (9–18 years old). Three patients were in the age group 2–5 years and 12 patients were in the age group 4. 

**Urine collection**

Random urine specimens were used for the Berry spot test and the DMB assay. Samples from early morning are not good specimens, because of higher excretion of GAGs than creatinine. During the day, the GAG:creatinine ratio remains constant (19). The urine samples were collected in sterile bottles without any preservatives and the physical, chemical, and microscopic properties of the urine were recorded by general urine analysis examination then stored at -80°C pending analysis.

**Urine sediment analysis**

A sample of 10–15 ml well-mixed urine was centrifuged in a test tube at 2500 rpm for 5–10 minutes. The supernatant was decanted and the sediment was resuspended in the remaining supernatant by flicking the bottom of the tube several times. A drop of resuspended sediment was poured onto a glass slide and coverslipped. The sediment was first examined for crystals, casts, squamous cells, and other large objects under low power to identify most. Several fields were averaged and the number of these elements reported as the number for each type found per low power field (LPF). Next, the examination was carried out at a high power to identify crystals, cells, and bacteria. The various types of cells were usually described as the number of each type found per average high power field (HPF) (20, 21).

**Spot test**

Urine samples were first evaluated for urinary excretion of mucopolysaccharides by Berry spot test using a toluidine blue dye and positive results double-checked. Briefly, 50 μl urine sample is spotted onto filter paper strips (Whatman 903 paper, Whatman, Dassel Germany); the paper is then dipped in toluidine blue dye solution (Merck, Germany) for 2 min. Subsequently, the paper is dried and the excessive dye is removed by washing with 1.8 M acetic acid and demineralized water. Positive samples will become purple against a bluish background (16, 22). 

**Quantitative measurement of urinary GAGs**

Urine creatinine was measured with commercial enzymatic kits (Pars Azmoon, Tehran, Iran), according to the manufacturer’s instructions. 1,9-dimethylene blue was purchased from Sigma-Aldrich (Sigma, Missouri, USA). Urinary GAGs was measured calorimetrically according to the previously described method (23). 

Concisely, based on reaction of DMB with urinary mucopolysaccharides, the complex was quantified spectrophotometrically in 520nm after 3 min. Results were expressed in mg/g creatinine.

**Statistical analysis**

Data was analyzed with SPSS 16 (SPSS Inc., Chicago). Results were presented as Mean±SD and independent samples T-test were used to compare mean differences. Moreover, a one-way ANOVA followed by a Post Hoc and Tukey and Dunnett tests were used to analyze differences between groups and a p value less than 0.05 was considered significant. Receiver operating characteristic (ROC) curves were constructed to establish a sensitivity-specificity relationship. Cut-off values that provided the best combination of sensitivity and specificity were determined by ROC curve analysis. The coefficient of variances of the test, sensitivity (true-positive/true-positive+false-negative), specificity (true-negative/true-negative+false-positive), positive predictive value (PPV, true-positive / true-positive + false-positive), negative predictive value (NPV, true-negative/true-negative+false-negative), positive likelihood ratio (LR+, sensitivity/1-specificity), and the negative likelihood ratio (LR-, 1-sensitivity / specificity) were calculated.

## Results

We have assessed the effects of urine properties on the Berry spot test. There was not any significant difference between urine characteristics with test results (Data not shown). For evaluation of those parameters that could result in erroneous outcomes in the DMB test, we distributed all samples into three groups based on the WBC cell counts in urine (Table 1). Total urinary glycosaminoglycans decreased in urine samples with a WBC cell count greater than 10 cells compared to samples with lower than 4 cells (Table 1). A similar alteration was also observed for urine epithelial cell counts: GAGs concentration being highest in Group 1 (samples with cell counts lower than 4 cells) and the lowest in Group 3 (samples with cell counts higher than 10 cells). On the other hand, despite apparent reductions in the GAGs level by the decline of the urine WBC and epithelial cell counts, a statistically significant correlation was not detected between Groups 2 and the other Groups (Table 1). Furthermore, we have evaluated the effects of other urine parameters on measurement of GAGs by the DMB colorimetric method. There was not any significant difference between other parameters such as urine pH, specific gravity (SG), and urine RBC with GAGs concentration. Furthermore, despite trends to decrease concentrations of urine GAGs with increasing urine protein; there was no significant difference between urine protein and GAGs (Data not shown).

**Fig 1:**
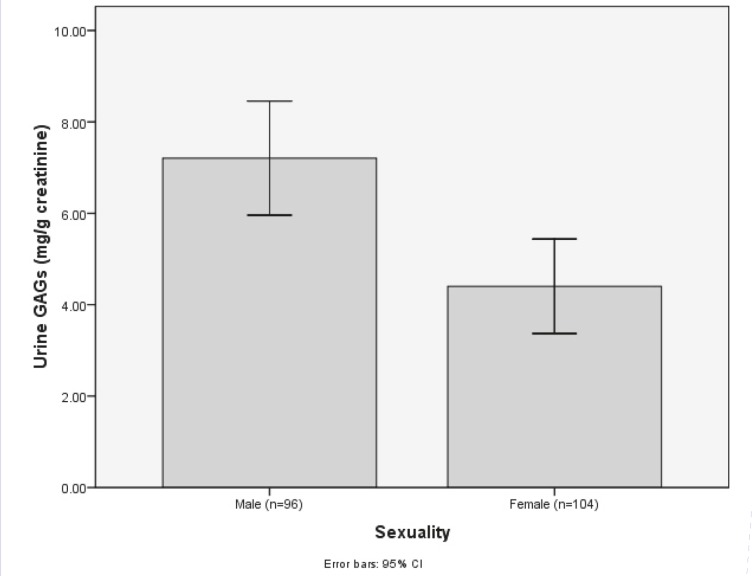
Concentrations of urinary GAGs according to gender

In addition, we evaluated the change of GAGs concentration in male and female subjects. Based on the DMB method, there was a significant differences between the urine GAGs in male and female subjects (Figure 1). Male subjects showed significantly (p<0.05) higher urine GAGs level than the female group did (7.21± 3.68 and 4.4 ± 3.44 mg/g creatinine, respectively). 

Furthermore, a One-Way ANOVA analysis followed by Tukey tests confirmed that urine GAGs was remarkably lower in Groups 4 and 5 of the age subclasses compared to other Groups (Table 1), According to age classification.

**Fig 2:**
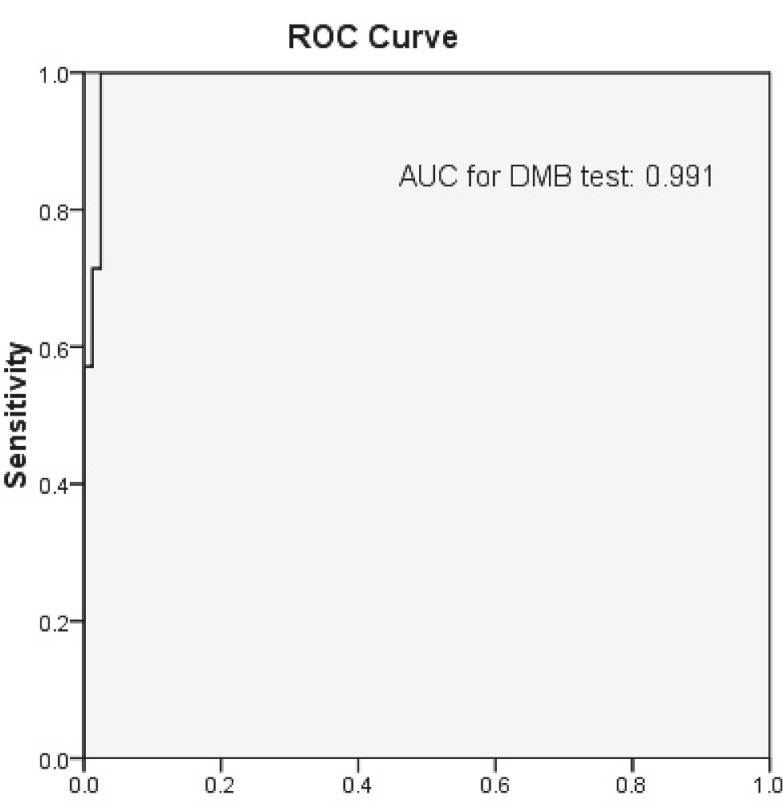
ROC curves for the DMB test. DMB = 1,9 Dimethylane blue; AUC = area under the curve; ROC = receiver operating characteristic

The sensitivity and specificity of the Berry spot test was 83.33% and 88.88%, respectively. Table 2 shows the other diagnostic utility for this test. The means and standard deviations of urinary GAGs for MPSI and control groups were 12.54 ± 2.14 mg/g creatinine and 4.67 ± 3.29 mg/g creatinine, respectively. Using a cut-off level of 10.73 mg/g creatinine for urinary GAGs, sensitivity, and specificity were 100% and 97.22%, respectively. The area under the curve for GAGs by Analysis of ROC curves (Figure 2) of the DMB test was 0.99, 95% CI=0.973-1. The positive predictive value (PPV), negative predictive value (NPV), positive likelihood ratio, and negative likelihood ratio of the tests were also determined. Table 2 shows the abovementioned values for urine samples of the studied subjects. The highest (97.22%) specificity and highest (0.75) PPV could be obtained by quantitative measurements of GAGs. Within run and between run CVs were obtained for the DMB test was 2% and 7%, respectively.

## Discussion

In the present study, we assessed the diagnostic validity of total glycosaminoglycans concentrations in the urine of MPSI patients compared healthy subjects. The random urine specimens were used for the DMB and the Berry spot tests. There are many studies that use random urine (8, 19) for measurement, but 24 h urine samples have also been utilized (24). Mahalingam K et al (25) used random urine for measurement of urine GAGs in the concentration of GAGs in the control group and MPS suspected patients were 2.5±2.09 and 12.64±12.83 mg, respectively. Additionally, de Jong JG et al (26) also determined the urine GAGs in untimed samples. The concentration of this marker in the patient group ranged from 2.9±1.0 mg in 15–20 age years and 18.6±8.6 mg in 0–1 age years. On the other hand, Panin G et al (27), which used 24 h urine samples, reported that the concentration of GAGs in urine was 12.8±5.0 mg/24h for healthy subjects, but they concentrated urine GAGs by cetylpyridinium chloride. There are no reported discrepancies between the results of two different sample collections; however, due to the age of the MPSI patients it seems that random specimens are a better choice for the assay.

We have evaluated the general properties of urine with the DMB and the Berry spot test results. Urine pH, SG, protein, WBC, epithelial cell, and bacteria count have no significant reducing or increasing effect on the Berry spot test. The Berry spot test provides a rapid, simple, and cheap qualitative evaluation of urine; however, the low sensitivity and specificity have precluded its use as a screening test (26, 28). Alternative spot tests also dealt with the same disadvantages for false-negative and false-positive specimens (18, 29, 30). De jong et al (23) has shown that azure A dye based spot test (Ames spot test) has a low reliability, with approximately 35% of false-negative and 29% of false-positive results. The Berry spot test sensitivity and specificity in our study was 83.33% and 88.88%, respectively. The most logical explanation for these results could be related to the studied population. In our study, we assessed the MPSI patients, whereas in other studies, all MPS patients were assessed. 

In this study, the mean concentration of urinary GAGs in MPSI patients and control subjects was 12.54 ± 2.14 mg/g creatinine and 4.67 ± 3.29 mg/g creatinine, respectively. In a study that was carried out in Chile, the median concentration of urinary GAGs/creatinine ratio in MPSI patients was 2.83 (24). In another study in Taiwan, the concentrations were 39.6 ± 11.8 mg/mmol creatinine for the patients group (31). However, Gallegos-Arreola et al (32) found that the highest levels of GAGs/creatinine was in patients with Hurler syndrome (mean=627.7). A comparison of these results revealed that there are significant differences between GAGs concentrations in different populations. This alteration could be due to sampling and/or patient selection and analysis methods. 

Although, due to lack of of patients in some age ranges, an appropriate interpretation of age and urine GAGs interactions could not be understood, our results revealed that there is an indirect correlation between urinary GAGs concentration and age; with increased age, the mean concentration of GAGs decreased. These results are supported by previous findings that revealed that urine GAGs concentrations have a negative correlation with age (6, 14, 24, 30, 32). A similar direct correlation was also observed between urinary GAGs and urine WBC and epithelial cell counts. Previous studies have demonstrated some urine properties on colorimetric assays of the GAGs. For example, many studies have proved the interference of urine proteins on the DMB assay (26, 33). We reported the negative effects of urine WBC and epithelial cell counts on the DMB assay for the for variations in urine concentrations by reference to specific gravity or creatinine concentrations. It is also recommended that specimens should not be from an early morning collection, have creatinine concentrations less than 1.5 mmol/L, SG lower than 1.008, and should not be infected (8, 17). In addition, our results clearly show that the concentration of urine GAGs in male subjects is higher than for females. 

We showed high sensitivity and specificity of urinary GAGs test based on DMB colorimetric assays. This finding is similar to that reported by previous studies.

However, in this study we demonstrate that high sensitivity and specificity of the GAGs assay in MPSI patients. In the present study, the sensitivity and specificity of the test was 100% and 97.22% for the DMB assay, respectively. Sensitivity and specificity reported by Mabe et al for the DMB test was 100% and 74.5%, respectively (24). It is the highest value that is reported for this test. Previous studies have shown that the sensitivity and specificity for the DMB test ranged from 96–100% and 94–100%, respectively (31, 33, 34). These findings show that the overall specificity of test is high and that this test has a low number of false-positive results, and could mitigate the disease in healthy subjects. In addition, because of the high sensitivity of the test, it rarely misses true positives among those who are actually positive.

In summary, we have shown the high sensitivity and specificity of the DMB test for determination of urine GAGs. In addition, our results also show an opposite correlation between the ages of patients with concentrations of urinary GAGs. Nevertheless, the results of the present study reveal that, some urine characteristics such as urine WBC and epithelial cell count might result in false-negative results. 

It can be concluded that the determination of urinary GAGs might be a simple, rapid, and inexpensive diagnostic tool for screening and monitoring of MPSI patients before using costly, laborious, and time-consuming tests, such as enzyme and molecular genetic assays.

## Notes

**Potential conflict of interest:** None declared.

## References

[1] Jackson RL, Busch SJ, Cardin AD (1991). Glycosaminoglycans: molecular properties, protein interactions, and role in physiological processes. Physiological reviews.

[2] Ghaderi S (2006). The biochemistry base of mucopolysaccharidoses and approach to Genetics in the 3rd millennium. [Educational].

[3] Mizumoto S, Ikegawa S, Sugahara K (2013 ). Human genetic disorders caused by mutations in genes encoding biosynthetic enzymes for sulfated glycosaminoglycans. The Journal of biological chemistry.

[4] Salbach J, Rachner TD, Rauner M, Hempel U, Anderegg U, Franz S (2012 ). Regenerative potential of glycosaminoglycans for skin and bone. Journal of molecular medicine (Berlin, Germany).

[5] Coppa GV, Catassi C, Gabrielli O, Giorgi PL, Dall’Amico R, Naia S (1987). Clinical application of a new simple method for the identification of mucopolysaccharidoses. Helvetica paediatrica acta.

[6] Fuller M, Meikle PJ, Hopwood JJ (2004). Glycosaminoglycan degradation fragments in mucopolysaccharidosis I. Glycobiology.

[7] Fuller M, Rozaklis T, Ramsay SL, Hopwood JJ, Meikle PJ (2004). Disease-specific markers for the mucopolysaccharidoses. Pediatric research.

[8] Blau N, Duran M, Gibson K Laboratory Guide to the Methods in Biochemical Genetics.

[9] Dorfman A, Matalon R (1969). The Hurler and Hunter syndromes. The American journal of medicine.

[10] Fratantoni JC, Hall CW, Neufeld EF (1968). Hurler and Hunter syndromes: mutual correction of the defect in cultured fibroblasts. Science (New York, NY).

[11] Fratantoni JC, Hall CW, Neufeld EF (1969). The defect in Hurler and Hunter syndromes. II. Deficiency of specific factors involved in mucopolysaccharide degradation. Proceedings of the National Academy of Sciences of the United States of America.

[12] Fratantoni JC, Neufeld EF, Uhlendorf BW, Jacobson CB (1969). Intrauterine diagnosis of the hurler and hunter syndromes. The New England journal of medicine.

[13] Chamoles NA, Blanco MB, Gaggioli D, Casentini C (2001). Hurler-like phenotype: enzymatic diagnosis in dried blood spots on filter paper. Clinical chemistry.

[14] Nor A, Zabedah MY, Norsiah MD, Ngu LH, Suhaila AR (2010). Separation of sulfated urinary glycosaminoglycans by high-resolution electrophoresis for isotyping of mucopolysaccharidoses in Malaysia. The Malaysian journal of pathology.

[15] De Muro P, Faedda R, Formato M, Re F, Satta A, Cherchi GM (2001). Urinary glycosaminoglycans in patients with systemic lupus erythematosus. Clinical and experimental rheumatology.

[16] Berry HK, Spinanger J (1960). A paper spot test useful in study of Hurler’s syndrome. The Journal of laboratory and clinical medicine.

[17] Pennock CA, White F, Murphy D, Charles RG, Kerr H (1973). Excess glycosaminoglycan excretion in infancy and childhood. Acta paediatrica Scandinavica.

[18] Berman ER, Vered J, Bach G (1971). A reliable spot test for mucopolysaccharidoses. Clinical chemistry.

[19] Pennock CA (1976). A review and selection of simple laboratory methods used for the study of glycosaminoglycan excretion and the diagnosis of the mucopolysaccharidoses. Journal of clinical pathology.

[20] Chan RW, Szeto CC (2004). Advances in the clinical laboratory assessment of urinary sediment. Clinica chimica acta; international journal of clinical chemistry.

[21] Fogazzi GB, Garigali G (2003). The clinical art and science of urine microscopy. Curr Opin Nephrol Hypertens.

[22] Berry HK (1987). Screening for mucopolysaccharide disorders with the Berry spot test. Clinical biochemistry.

[23] de Jong JG, Hasselman JJ, van Landeghem AA, Vader HL, Wevers RA (1991). The spot test is not a reliable screening procedure for mucopolysaccharidoses. Clinical chemistry.

[24] Mabe P, Valiente A, Soto V, Cornejo V, Raimann E (2004). Evaluation of reliability for urine mucopolysaccharidosis screening by dimethylmethylene blue and Berry spot tests. Clinica chimica acta; international journal of clinical chemistry.

[25] Mahalingam K, Janani S, Priya S, Elango EM, Sundari RM (2004). Diagnosis of mucopolysaccharidoses: how to avoid false positives and false negatives. Indian J Pediatr.

[26] de Jong JG, Wevers RA, Laarakkers C, Poorthuis BJ (1989). Dimethyl methylene blue-based spectrophotometry of glycosaminoglycans in untreated urine: a rapid screening procedure for mucopolysaccharidoses. Clinical chemistry.

[27] Panin G, Naia S, Dall’Amico R, Chiandetti L, Zachello F, Catassi C (1986). Simple spectrophotometric quantification of urinary excretion of glycosaminoglycan sulfates. Clinical chemistry.

[28] Byers S, Rozaklis T, Brumfield LK, Ranieri E, Hopwood JJ (1998). Glycosaminoglycan accumulation and excretion in the mucopolysaccharidoses: characterization and basis of a diagnostic test for MPS. Molecular genetics and metabolism.

[29] Carson NA, Neill DW (1962). Metabolic abnormalities detected in a survey of mentally backward individuals in Northern Ireland. Archives of disease in childhood.

[30] Huang KC, Sukegawa K, Orii T (1985). Screening test for urinary glycosaminoglycans and differentiation of various mucopolysaccharidoses. Clinica chimica acta; international journal of clinical chemistry.

[31] Chih-Kuang C, Shuan-Pei L, Shyue-Jye L, Tuen-Jen W (2002). MPS screening methods, the Berry spot and acid turbidity tests, cause a high incidence of false-negative results in sanfilippo and morquio syndromes. Journal of clinical laboratory analysis.

[32] Gallegos-Arreola MP, Machorro-Lazo MV, Flores-Martinez SE, Zuniga-Gonzalez GM, Figuera LE, Gonzalez-Noriega A (2000). Urinary glycosaminoglycan excretion in healthy subjects and in patients with mucopolysaccharidoses. Archives of medical research.

[33] Piraud M, Maire I, Mathieu M (1993). Pitfalls of screening for mucopolysaccharidoses by the dimethylmethylene blue test. Clinical chemistry.

[34] Whitley CB, Spielmann RC, Herro G, Teragawa SS (2002). Urinary glycosaminoglycan excretion quantified by an automated semimicro method in specimens conveniently transported from around the globe. Molecular genetics and metabolism.

